# Single Production of Kojic Acid by *Aspergillus flavus* and the Revision of Flufuran

**DOI:** 10.3390/molecules24224200

**Published:** 2019-11-19

**Authors:** Antonius R. B. Ola, Gema Metboki, Caterina S. Lay, Yoseph Sugi, Philipi De Rozari, Dodi Darmakusuma, Euis Holisotan Hakim

**Affiliations:** 1 Chemistry Department, Faculty of Science and Engineering, Nusa Cendana University, Kupang 85001, Indonesia; gemametboki18@gmail.com (G.M.); caterina.lay11@gmail.com (C.S.L.); tonc2k@yahoo.com (Y.S.); phderozari@yahoo.com (P.D.R.); dodikimia@yahoo.com (D.D.); 2 Integrated Research Center Laboratory (UPT LRT Biosains), Nusa Cendana University 2, Kupang 85001, Indonesia; 3 Department of Chemistry, Institute of Technology Bandung, Jl. Ganesha 10, Bandung 40132, Indonesia; euis@chem.itb.ac.id

**Keywords:** timor, kojic acid, endophytic fungi, catharanthus roseus, annona squamosa, curcuma xanthorisa, sonneratia alba

## Abstract

Timor Island is very hot and dry due to the high intensity of sunlight experienced throughout the year. The endophytic fungi *Aspergillus flavus* had been isolated from medicinal plants such as *Catharanthus roseus*, *Annona squamosa* and *Curcuma xanthorisa*. The endophytic fungi *A. flavus* from each plant was cultivated on solid rice media and then analyzed for its capability for producing kojic acid. The production of kojic acid was analyzed by HPLC; the highest amount of kojic acid was observed from the endophytic fungi *A. flavus,* isolated from the stem of *Catharanthus roseus,* followed by *A. flavus* from *Annona squamosa* and *Curcuma xanthorisa*. Simple VLC fractionation of the extract of *A. flavus* from *C.roseus* led to the isolation of around 11.1 g of pure kojic acid. The structure of kojic acid (**1**) was confirmed by NMR and MS spectroscopic data. A comparison of the NMR data with the literature supported the revision of the natural product flufuran to kojic acid. To the best of our knowledge, this is the first report of a strain of endophytic fungi producing only kojic acid without any other toxic metabolites such as alfatoxins. Therefore, this *Aspergillus flavus* strain can be applied as a potential producer of kojic acid for industrial use.

## 1. Introduction

Endophytic fungi have been known as great sources of bioactive, secondary metabolites with potential application in medicine, agriculture, and the pharmaceutical industry. One of the most economically important drugs from endophytic fungi is a blood cholesterol lowering agent known as “statin”, for which mevastatin and lovastatin are produced by the endophytic fungi *Penicilium citrinum* and *Aspergillus terreus*.

Besides producing new compounds, endophytic fungi have been well recorded to produce the same metabolites from plants. For example, the most effective anticancer drugs such as vinca alkaloids vincristine and vinblastine, paclitaxel, and campthothecin have been known to be produced by endophytic fungi [1]. Thus, endophytic fungi hold great promise in the search for bioactive agents, as they also need only a small amount of material from plants. As part of our ongoing investigation on biologically-active, natural products from endophytic fungi, we have isolated kojic acid from endophytic fungi *Aspergillus flavus* from several medicinal plants growing in Timor Island, Eastern Indonesia. High exposure to sunlight can lead to an increased risk of several types of damage to human skin such as sunburn, skin cancer, and oxidative stress [2]. To prevent the harmful effects of ultraviolet radiation, kojic acid has been incorporated into various cosmetic formulations such as creams, lotions, and soaps due to its ability to inhibit hyperpigmentation tyrosinase. Besides that, it is also used to prevent food browning [3]. Its potential use and wide application in the cosmetics and personal care, food, agricultural, pharmaceutical, medicine, and chemical industries have generated high demand of this metabolite and its derivatives [3]. Kojic acid is commonly produced by *Aspergillus flavus* and *Aspergillus oryzae*. However, organisms producing kojic acid also make other products, such as aflatoxin and cyclopiazonic acid [4]. The cooccurrence of kojic acid and aflatoxin has been known since 1966 [5]. More recently, 34 strains from Argentinean peanuts identified as *A. flavus* produced kojic acid, aspergillic acid, cyclopiazonic acid, aflatoxins B1 and B2, oryzaechlorin, and flavimine [6]. The same report also showed that two *Aspergillus* species section *flavi* from Argentina, i.e., *Aspergillus arachidicola* and *Aspergillus minisclerotigenes*, produced aflatoxins B1, B2, G1, G2, cyclopiazonic acid, and kojic acid [6]. *Aspergillus pseudocaelatus* is also known to produce alfatoxins, cyclopiazonic acid, and kojic acid [7]. Other reports showed that *A. flavus* produced both alfatoxins and kojic acid [8], while some only produced alfatoxins [9]. In this study, we report the production of a cosmetic compound only, i.e., kojic acid, by *Aspergillus flavus* isolated from Timorese medicinal plants. Using this source, other common metabolites of *Aspergilli* such as aflatoxins and cylopiazonic acid were not detected.

## 2. Results and Discussion

**Figure 1:**
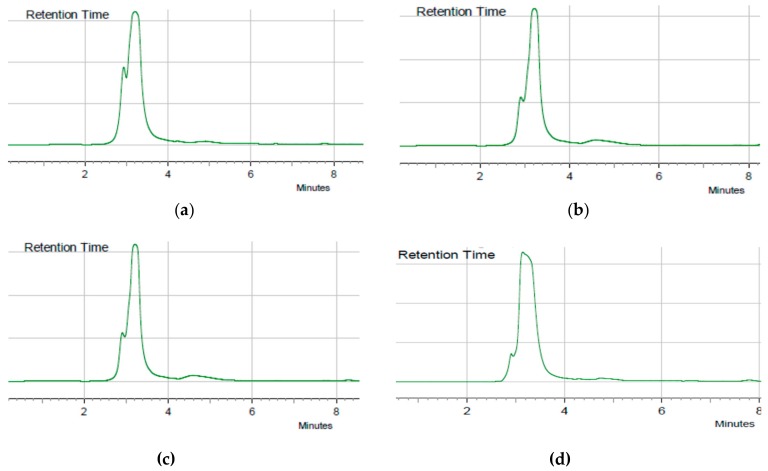
HPLC Chromatograms of extracts *from Aspergillus flavus* strain isolated from (**a**) Catharanthus roseus, (**b**) Annona squamosa, (**c**) Curcuma xanthoriza, and (**d**) Isolated kojic acid (**1**).

Endophytic fungi *Aspergillus flavus* were isolated from the medicinal plants *A. squamosa*, *C. roseus,* and *C. longa* that were growing in the same area (Dusun Binilaka, Kupang). Each endophytic fungus was cultured on 100 g solid rice media in 1 L Erlenmeyer flasks for 3-4 weeks. The culture of each fungus was extracted with ethyl acetate, yielding 18.8673 g, 12.4966 g, and 18.3599 g extracts of *A. flavus* isolated from *C. roseus*, *C. longa,* and *A. squamosa*, respectively. Each ethyl acetate crude extract was analyzed with HPLC to analyze the chemical metabolite content; they were also evaluated for their production of kojic acid (Figure 1). Based on the results from the HPLC analysis, *Aspergillus flavus* from *C. roseus*, *C. longa,* and *A. squamosa* were found to produce kojic acid, with the highest content observed from the *A. flavus* strain from *C. roseus*. The extract of endophytic fungi *A. flavus* from *C. roseus* was further subjected to fractionation using vacuum liquid chromatography (VLC). Each fraction of VLC was monitored by HPLC, and fractions 7–9 showed the same single peak as observed in the crude extract (11.1 g).

**Figure 2:**
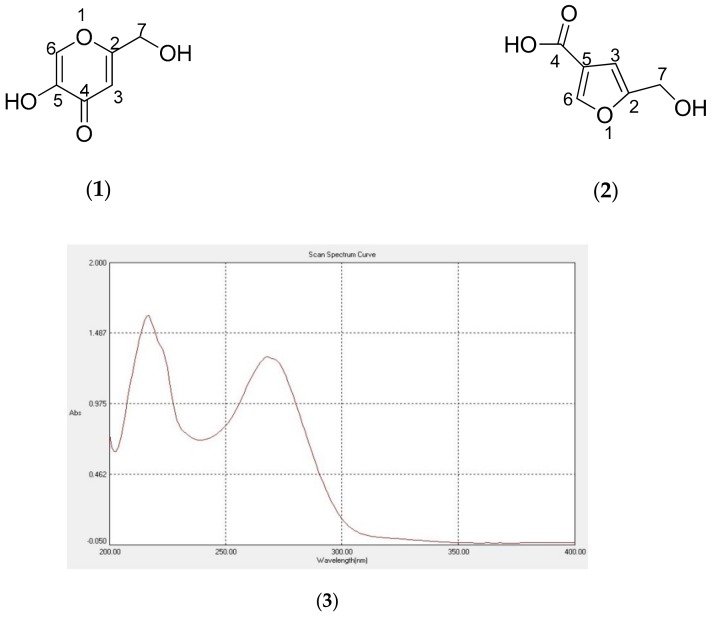
Chemical structures of (**1**) kojic acid and (**2**) flufuran, and the (**3**) UV Spectra of **1**.

The obtained pure compound (**1**) showed a molecular ion peak at 143.0335 [M + H]^+^ by HR ESIMS. The ^1^H-NMR and ^13^C-NMR spectra (Table 1) were identical to those reported for flufuran [10,11,12]. However, flufuran was first reported not as a natural product but as a synthetic intermediate [13]. Flufuran was identified as a natural product for the first time from the fungal culture *Polyporus ciliatus* by Cabreca et al. in 2002 [10]. Afterwards, it was reported from several fungi by referring to the first report from Cabreca et al. in 2002 [10]. A comparison of the NMR data between flufuran reported as a natural product [10,11] and as a synthetic intermediate [14] showed several discrepancies. The NMR data reported for flufuran as a natural product were identical to those reported as kojic acid, but significantly different with synthetic flufuran (Table 1). In addition, the UV spectra of flufuran in the first report of its isolation as a natural product by Cabreca et al. in 2002 was also identical to the UV spectra of kojic acid, showing λmax at 217 and 269 nm in methanol (Figure 2) [10]. As the NMR and UV data of flufuran were the same as kojic acid, flufuran previously isolated from fungi *Polyporus ciliates* and *Aspergillus flavus* should be assigned as kojic acid. As compound **1** was isolated from *Aspergillus flavus,* which was well known as a producer of kojic acid together with the identical NMR and UV spectral data, compound **1** was then determined to be kojic acid.

## 3. Discussion

The first identification of flufuran as a natural product from the fungus *P. ciliatus* referred to the synthetic product made by Pevzner et al. in 1999 and by Cabreca et al. in 2002 [10]. The second report of flufuran as a natural product from the strain of *Aspergillus flavus* was authored by Evidenti et al. in 2009 [11], referring to an interpretation of the work by Cabreca et al. in 2002 [10]. Other methods of production of flufuran were also reported with *Aspergillus flavus* [12,15]. Due to the fact *Aspergillus flavus* is well known as a producer of kojic acid [4], and given that the first report of natural flufuran [10] was in reference to a synthetic intermediate [13], together with inaccurately-assigned NMR data (Table 1), this led to the misinterpretation of flufuran instead of kojic acid from several fungi [10,11,12,15,16,17]. This is in agreement with a recent report by DellaGreca et al. [14], i.e., that flufuran was misidentified, and thus, all previous reports describing the isolation and the bioactivities of flufuran from fungi should be interpreted as representing kojic acid. Kojic acid was commonly produced by *Aspergillus* species, together with other metabolites [4]. It is interesting to note that this strain of *Aspergillus flavus* produced only kojic acid. Alfatoxins and other common metabolites of *Aspergilli* such as cylopiazonic acid were not detected. To the best of our knowledge, this is the first report of the production of only kojic acid without other toxic metabolites from the species *Aspergillus flavus* and/or *Aspergillus oryzae*. The species of *Aspergillus flavus* from Timor, Indonesia, resembles that which Hink isolated from a chestnut orchard in Caserta, Italy, which produced only one main metabolite and no alfatoxins [11].

As all the plant materials were collected from the same location which is hot and very dry due to the high intensity of sunlight throughout the year, the production of kojic acid by *Aspergillus flavus* was probably as a response to stress caused by this ecological condition.

## 4. Materials and Methods

### 4.1. Isolation of Endophytic Fungi

Fresh and healthy samples of *Annona squamosa, Catharanthus roseus,* and *Curcuma longa* were collected in Kupang, East Nusa Tenggara Province, Indonesia. The methods of isolation and identification of endophytic fungi were consistent with those previously described [18,19].

### 4.2. Cultivation and Extraction of Secondary Metabolites

Each endophytic fungi of *Aspergillus flavus* from *A. squamosa*, *C. roseus,* and *C. longa* was cultivated in two Erlenmeyer flasks (1 L). A quarter of pure culture grown on PDA was added into to rice media (100 g of rice in 110 mL of distilled) which had been autoclaved. The rice medium containing the fungus was cultivated under static conditions at room temperature for 3–4 weeks. After that, ethyl acetate (250 mL) was added into Erlenmeyer flasks and kept overnight. The following day, the culture was filtered and the ethyl acetate was removed under a vacuum.

### 4.3. Analysis of Kojic Acid with HPLC

Five mg of ethyl acetate extract of each endophytic fungi *Aspergillus flavus* was dissolved in 1 mL of methanol. From the solution, 20 µL was injected into the column (ACE C18 150 × 4.6 m) at a flow rate 0.5 mL/min, with detection at 204 nm, using methanol as the mobile phase. The standard of kojic acid was measured with a concentration of 1 mg/mL, with the same volume of injection, i.e., 20 µL.

### 4.4. Fractionation and Identification of Kojic Acid

The ethyl acetate crude extract of *Aspergillus flavus* (25 g) was subjected to fractionation through vacuum liquid chromatography (VLC) using mixtures of solvent with gradient polarity starting from n-hexane-EtOAc, followed by CH_2_Cl_2_-MeOH. Fraction 7–9 eluted with ethyl acetate and DCM: MeOH yielded pure compound **1** (11.1gram).

The structure of compound **1** was identified using the art of 1D and 2D NMR and mass spectral data. NMR data were recorded using 500 MHz for ^1^H and 125 MHz for ^13^C. Methanol (MeOD) was used as solvent, see Supplementary Materials.

## Figures and Tables

**Table 1:** ^1^H-NMR (500 MHz) dan ^13^C-NMR (125 MHz) Data for kojic acid and flufuran in CD_3_OD.

No	Kojic Acid (1)		Flufuran Natural Products ^a,b^				Flufuran Synthetic ^c^	
	^1^H-NMR	^13^C-NMR	^1^H-NMR^a^	^13^C-NMR ^a^	^1^H-NMR ^b^	^13^C-NMR ^b^	^1^H-NMR	^13^C-NMR
1								
2	-	170.41		170.4		170.4		156.4
3	6.49 s, 1H	110.74	6.53	110.7	6.49	110.8	6.62	106.7
4	-	176.86		176.9		176.9		165.1
5	-	147.37		147.5		147.4		120.1
6	7.95 s, 1H	141.0	7.98	141.0	7.94	141.0	8.08	147.7
7	4.40 s, 2H	61.18	4.43	61.2	4.4	61.2	4.53	55.7

^a^ Evidenti et al., 2009; ^b^ Cabreca et al., 2002; ^c^ DellaGreca et al., 2019.

## Supplementary Materials

The Supplementary Materials of ^1^H, ^13^C NMR and DEPT Spectra are available online.
