# Supplementary material for: Single Production of Kojic Acid by Aspergillus flavus and the Revision of Flufuran
Source: Molecules. 2019 Nov 19;24(22):4200. doi: 10.3390/molecules24224200 (PMC6891502; doi:10.3390/molecules24224200)

# Supplementary Materials

## Single Production of Kojic Acid by *Aspergillus flavus* and the Revision of Flufuran

**Antonius R B Ola [1,2,*], Gema Metboki [1], Catherine D Lay[1], Yoseph Sugi[1], Philipi De Rozari[1], Dodi Dharmakusuma[1,2] and Euis Holisotan Hakim[3]**

1 Chemistry Department, Faculty of Science and Engineering, Nusa Cendana University, Kupang, Indonesia; antonius.ola@staf.undana.ac.id
2 Integrated Research Center Laboratory (Biosains), University of Nusa Cendana 2; ola.antonius@gmail.com
3 Department of Chemistry, Institute of Technology Bandung, Jl. Ganesha 10, Bandung 40132
* Correspondence: antonius.ola@staf.undana.ac.id

## Table of Contents


$^{1}$H NMR spectrum of **1** in methanol-*d*$_4$ ........................................................................................3

$^{13}$C NMR spectrum of **1** in methanol-*d*$_4$ ......................................................................................4

DEPT spectrum of **1** in methanol-*d*$_4$...............................................................................................5

**Figure S1.** $^{1}H$ NMR spectrum of **1** (methanol-$d_4$)

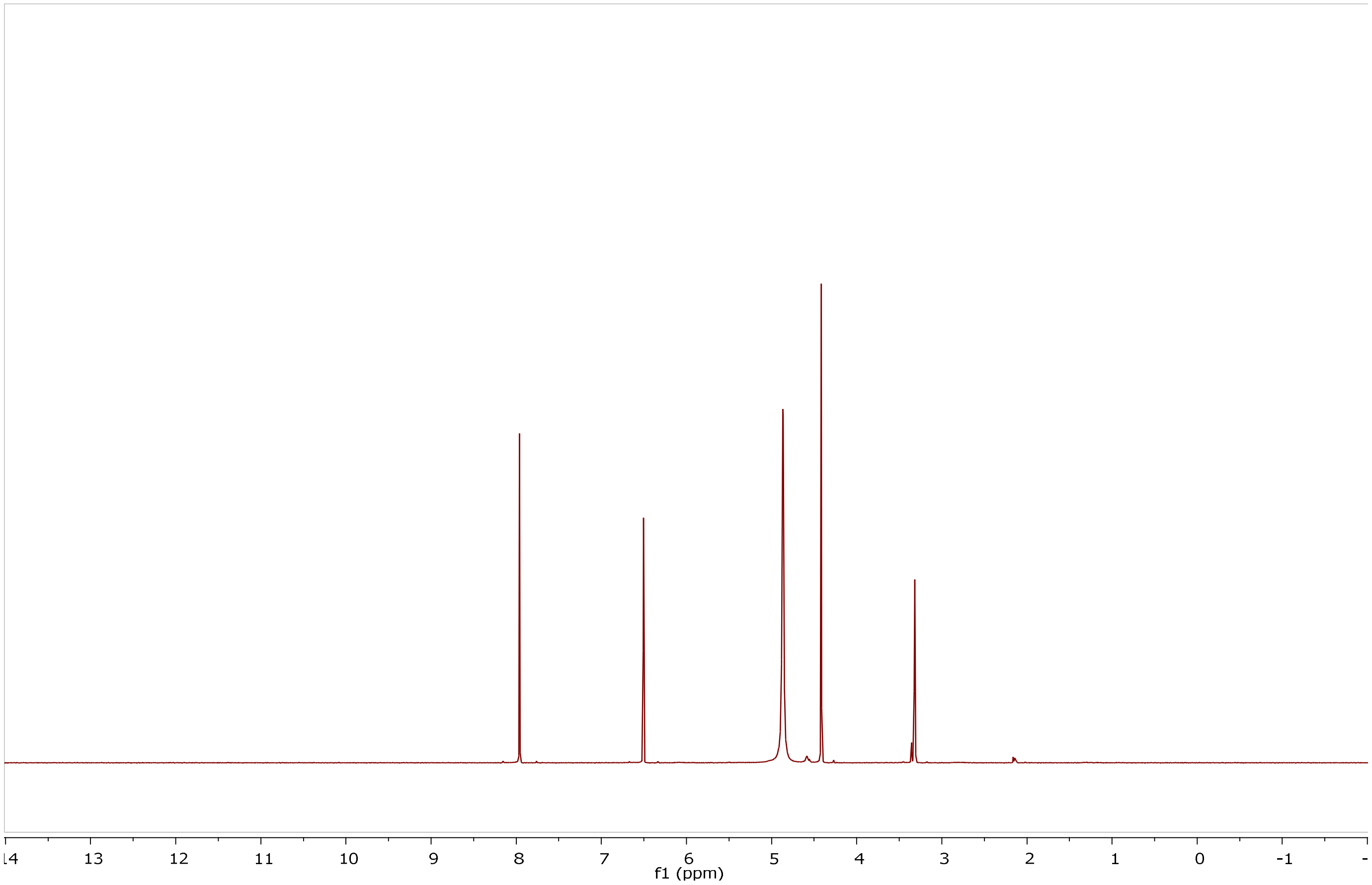

**Figure S2.** $^{13}C$ NMR spectrum of **1** (methanol-$d_4$)

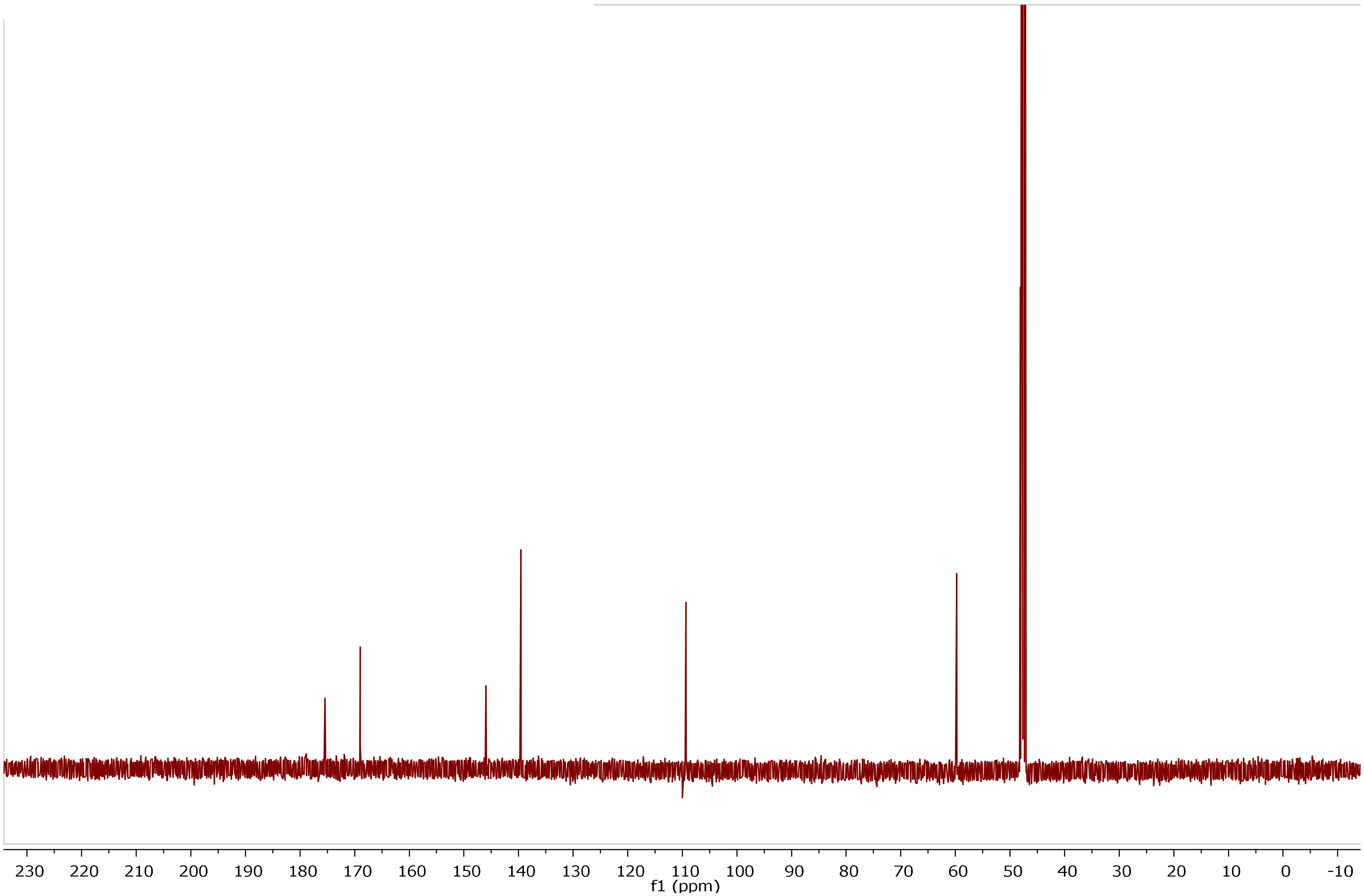

**Figure S3.** DEPT spectrum of **1** (methanol-$d_4$)

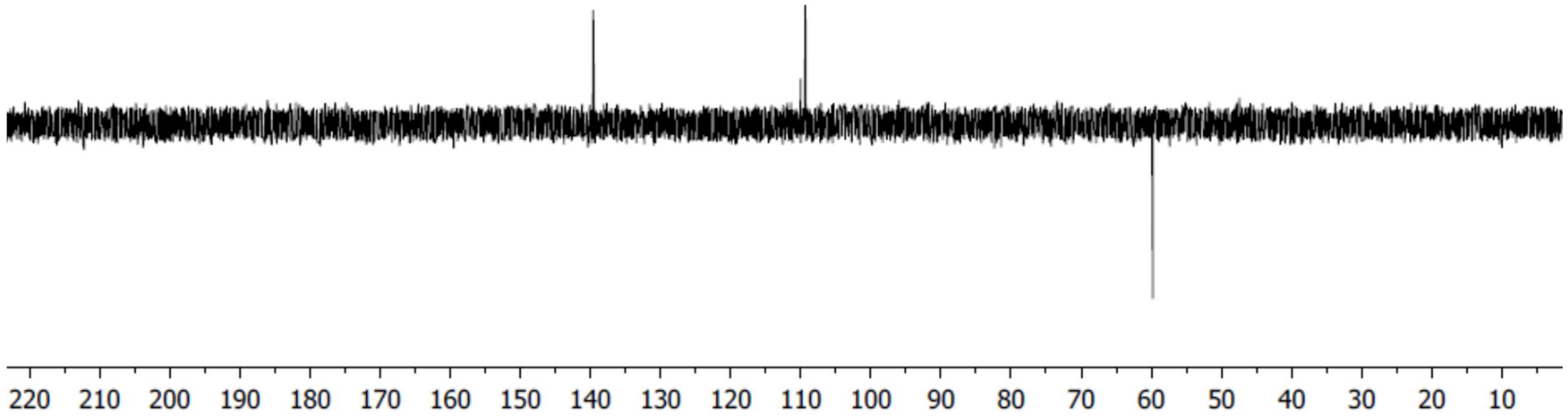

Supplement: Supplementary file 1 [file molecules-24-04200-s001.pdf]
